# Identification of Peptide Inhibitors of Enveloped Viruses Using Support Vector Machine

**DOI:** 10.1371/journal.pone.0144171

**Published:** 2015-12-04

**Authors:** Yongtao Xu, Shui Yu, Jian-Wei Zou, Guixiang Hu, Noorsaadah A. B. D. Rahman, Rozana Binti Othman, Xia Tao, Meilan Huang

**Affiliations:** 1 School of Chemistry and Chemical Engineering, Queen's University Belfast, David Keir Building, Stranmillis Road, Belfast, Northern Ireland, United Kingdom; 2 School of Basic Medical Sciences, Xinxiang Medical University, Xinxiang, Henan, China; 3 School of Biotechnology and Chemical Engineering, Ningbo Institute of Technology, Zhejiang University, Ningbo, China; 4 Department of Chemistry, Faculty of Sciences, University of Malaya, Kuala Lumpur, Malaysia; 5 Drug Design & Development Research Group, University of Malaya, Kuala Lumpur, Malaysia; 6 Department of Pharmacy, Faculty of Medicine, University of Malaya, Kuala Lumpur, Malaysia; 7 State Key Laboratory of Organic-Inorganic Composites, Beijing University of Chemical Technology, Beijing, China; Second University of Naples, ITALY

## Abstract

The peptides derived from envelope proteins have been shown to inhibit the protein-protein interactions in the virus membrane fusion process and thus have a great potential to be developed into effective antiviral therapies. There are three types of envelope proteins each exhibiting distinct structure folds. Although the exact fusion mechanism remains elusive, it was suggested that the three classes of viral fusion proteins share a similar mechanism of membrane fusion. The common mechanism of action makes it possible to correlate the properties of self-derived peptide inhibitors with their activities. Here we developed a support vector machine model using sequence-based statistical scores of self-derived peptide inhibitors as input features to correlate with their activities. The model displayed 92% prediction accuracy with the Matthew’s correlation coefficient of 0.84, obviously superior to those using physicochemical properties and amino acid decomposition as input. The predictive support vector machine model for self- derived peptides of envelope proteins would be useful in development of antiviral peptide inhibitors targeting the virus fusion process.

## Introduction

Fusion process is the initial step of viral infection, therefore targeting the fusion process represents a promising strategy in design of antiviral therapy [1]. The entry step involves fusion of the viral and the cellular receptor membranes, which is mediated by the viral envelope (E) proteins. There are three classes of envelope proteins [2]: Class I E proteins include influenza virus (IFV) hemagglutinin and retrovirus Human Immunodeficiency Virus 1 (HIV-1) gp41; Class II E proteins include a number of important human flavivirus pathogens such as Dengue virus (DENV), Japanese encephalitis virus (JEV), Yellow fever virus (YFV), West Nile virus (WNV), hepatitis C virus (HCV) and Togaviridae virus such as alphavirus Semliki Forest virus (SFV); Class III E proteins include vesicular stomatitis virus (VSV), Herpes Simplex virus-1 (HSV-1) and Human cytomegalovirus (HCMV). Although the exact fusion mechanism remains elusive and the three classes of viral fusion proteins exhibit distinct structural folds, they may share a similar mechanism of membrane fusion [3].

A peptide derived from a protein-protein interface would inhibit the formation of that interface by mimicking the interactions with its partner proteins, and therefore may serve as a promising lead in drug discovery [4]. Enfuvirtide (T20), a peptide that mimicks the HR2 region of Class I HIV-1 gp41, is the first FDA-approved HIV-1 fusion drug that inhibits the entry process of virus infection [5–7]. Then peptides mimicking extended regions of the HIV-1 gp41 were also demonstrated as effective entry inhibitors [8, 9]. Furthermore, peptides derived from a distinct region of GB virus C E2 protein were found to interfere with the very early events of the HIV-1 replication cycle [10]. Other successful examples of Class I peptide inhibitors include peptide inhibitors derived from SARS-CoV spike glycoprotein [11–13] and from Pichinde virus (PICV) envelope protein [14]. Recently, a peptide derived from the fusion initiation region of the glycoprotein hemagglutinin (HA) in IFV, Flufirvitide-3 (FF-3) has progressed into clinical trial [15].

The success of developing the Class I peptide inhibitors into clinical use has triggered the interests in the design of inhibitors of the Class II and Class III E proteins. e.g. several hydrophobic peptides derived from the Class II DENV and WNV E proteins exhibited potent inhibitory activities [16–20]. In addition, a potent peptide inhibitor derived from the domain III of JEV glycoprotein and a peptide inhibitor derived from the stem region of Rift Valley fever virus (RVFV) glycoprotein were reported [21, 22]. Examples of the Class II peptide inhibitors of enveloped virus also include those derived from HCV E2 protein [23, 24] and from Claudin-1, a critical host factor in HCV entry [25]. Moreover, peptides derived from the Class III HSV-1 gB also exhibited antiviral activities [26–31], as well as those derived from HCMV gB [32].

Computational informatics plays an important role in predicting the activities of the peptides generated from combinatorial libraries. *In silico* methods such as data mining, generic algorithm and vector-like analysis were reported to predict the antimicrobial activities of peptides [33–35]. In addition, quantitative structure-activity relationships (QSAR) [36–40] and artificial neural networks (ANN) were applied to predict the activities of peptides [41, 42]. Recently, a support vector machine (SVM) algorithm was employed to predict the antivirus activities using the physicochemical properties of general antiviral peptides [43]. However, the mechanism of action of antiviral peptides is different from antimicrobial peptides; in fact, various protein targets are involved in the virus infection. e.g. HIV-1 virus infection involves virus fusion, integration, reverse transcription and maturation, etc. Thus it is difficult to retrieve the common features from general antiviral peptides to represent their antiviral activities. Virus fusion is mediated by E proteins. Although E proteins are highly divergent in sequence and structure, they share a common pathway of membrane fusion dynamics. i.e. E proteins experience significant conformational change to form a-trimer-of-hairpin, which drives the fusion of viral membrane and host membrane [44]. The antiviral peptides derived from enveloped proteins function by *in situ* binding to their respective accessory proteins, disrupting forming of the trimer-of-hairpin and membrane fusion, and therefore inhibiting the virus infection. In view of the important role of E proteins in virus fusion process and common mechanism of action of self-derived peptides, we developed a SVM model to predict the antiviral activities of self-derived peptides using sequence-based statistical scores as input features. The sequence-based properties were calculated by a conditional probability discriminatory function which indicates the propensity of each amino acid for being active at a specific position. Our model exhibited remarkably higher accuracy in predicting the activities of self-derived peptides, compared to the previous models developed for general antiviral peptides using classical physicochemical properties as descriptors [43]. The method would be useful in identification of entry inhibitors as a new generation of antiviral therapies.

## Methods

### Data collection

202 peptide virus entry inhibitors of enveloped viruses were collected, among them, 101 are active peptides and 101 are non-active peptides. These peptides comprised the 75p+75n training set of SVM models. The remaining 26 active peptides and 26 non-active peptides inhibitors were used as the test set.

#### Amino acid composition

Amino acid composition is the fraction of each amino acid in a peptide. The fraction of the 20 amino acids was calculated using the following equation:

Fraction of amino acid X=Total number of X/peptide length

### Physicochemical properties

Five physicochemical properties were used in SVM models. Isoelectric point (PI), Molecular weight (MW) and Grand average of hydropathicity (GRAVY) [45] were calculated using the Protparam tool implemented in Expasy web server. Solvent accessibility and secondary structure features were calculated using SSpro and ACCpro packages implemented in the SCRATCH protein predictor server [46].

#### Sequence-based statistical scoring function

The knowledge-based statistical function is developed from the concept of residue-specific all-atom probability discriminatory function (RAPDF) [47]. RAPDF is a structure-based statistical scoring function. It is based on the assumption that averaging over different atom types in experimental conformations is an adequate representation of the random arrangements of these atom types in any compact conformation. Here we developed a sequence-based statistical scoring function, where we presume that averaging over different amino acid sequences with experimental validated inhibitive activities is an adequate representation of the random amino acid sequences with any inhibitory activity. The basis of this assumption is that the peptides share a common mechanism of action, i.e. the peptides derived from E proteins bind competitively to their partner proteins, disrupt the forming of a-trimer-of-hairpin, and therefore inhibit the virus membrane fusion.

The sequence-based scoring function is described in the following form:

$$S(\{q_{a}^{i}\})=-\ln\frac{P(q_{a}^{i}|C)}{P(q_{a}^{i})}$$ (1)

Here, $q_{a}^{i}\in \{active\}$.

$P(q_{a}^{i}|C)$ is the probability of observing amino acid *i* in an active peptide sequence;

$P(q_{a}^{i})$ is the probability of observing amino acid *i* in any peptide sequence, active or non-active. They are approximately estimated using the following forms:

$$P(q_{a}^{i}|C)\equiv \frac{N_{obs}(i,a)}{N_{obs}(a)}$$ (2)

$$P(q_{a}^{i})\equiv \frac{N_{obs}(i)}{N_{total}}$$ (3)

*N*_*obs*_(*i*,*a*): The number of observed amino acid *i* within active peptides.

*N*_*obs*_(*i*): The number of observed amino acid *i* within active peptides and non-active peptides.

*N*_*obs*_(*a*): The number of observed amino acid types within active peptides.

*N*_*total*_: The number of observed amino acid types within active peptides and non-active peptides.

Similarly, we employed a dataset of experimentally verified non-active peptides in developing the statistical function, where $q_{a}^{i}\in \{inactive\}$.

For a given amino acid sequence, 20 columns of input are generated, corresponding to the occurrence of twenty natural amino acids at each position. Each column is assigned a value of *N* * (−log–*likelihood*), where N is the number of amino acid and −log–*likelihood* is derived from the statistical function score. Each of the features thus combines the propensity of the amino acid for being active or non-active with the corresponding amino acid composition.

Below is an example of calculating the statistical scores for a given peptide sequence:

The amino acid order for SVM input features is set as:

ACDEFGHIKLMNPQRSTVWY.

If the amino acid sequence of an active peptide inhibitor is:

DCPNGPWVWVPAFCQAVGWG,

the statistical N values of the sequence would be:

2,2,1,0,1,3,0,0,0,0,0,1,3, 1,0,0,0,3,3,0

The scores in the statistical function library based on the active peptide inhibitors are decided by Eq (1): -0.0856, 0.5057, 0.4740, 0.4133, -0.0856, -0.0856, 0.6439, 0.2508, 0.9440, -0.4670, 1.8603, 0.1330, 0.2261, -0.0115, 0.2761, 0.3288, 0.0479, -0.1207, 0.0079, 0.6816,

Therefore, the 20 SVM input features for the sequence would be: -0.1712, 1.0114, 0.4740, 0, -0.2568, -0.2568, 0, 0, 0, 0, 0, 0.1330, 0.6783, -0.0115, 0, 0, 0, -.3621, 0.0237, 0.

### SVM Parameter Optimization

SVM models combined with radial basis function (RBF) kernel parameters were developed using the C-SVC module in LIBSVM (version 3.1) [48, 49] and executed under the Matlab interface. The performance of SVM depends on two parameters, gamma -*g* and cost–*c* [50]. The default value is 1 for -*c* and 1/k for -g, where *k* is the number of input entries. Various pairs of (*c*, *g*) values were converted to exponential values (i.e. 2^*x*^;2^*y*^) and optimized using cross-validation and the pair with the best cross-validation accuracy was selected.

5-fold cross validation was performed to evaluate the performance of SVM models. In the evaluation process, dataset was partitioned randomly into five equally sized subsets. The training and testing were carried out five times, each time four distinct subsets being used as training sets and the remaining subset as test set. The results were averaged over all five rounds of validation. The following equations were used to evaluate the prediction quality of the SVM models [48, 51]:

Sensitivity=[TP(TP+FN)]*100

Specificity=[TN(TN+FP)]*100

Accuracy=[(TP+TN)(TP+TN+FP+FN)]*100

$$MCC=\frac{TP*TN-FP*FN}{\sqrt{\left(TP+FP)*(TP+FN)*(TN+FP)*(TN+FN\right)}}$$

In the above equations, TP is the number of true positives, TN is the number of true negatives, FP is the number of false positives and FN is the number of false negatives. Matthew’s correlation coefficient (MCC) reflects the performance of the model. It ranges between -1 to 1 and a larger MCC value indicates a better prediction.

## Results and Discussion

SVM learning algorithm is a powerful machine learning method that has been widely used in pattern recognition and classification. SVM trains a dataset of experimentally validated positive and negative samples and generates a classifier to classify unknown samples into two distinct categories (positive or negative).

### Collection of dataset

We performed an exhaustive literature search on self-derived peptide inhibitors of enveloped proteins and collected experimentally validated peptides derived from the three classes of E proteins. For those peptides with overlapping segments, only one peptide sequence was kept. 202 peptides were found, among them, 101 are active peptides and 101 are non-active peptides (Table 1). 75 active peptide inhibitors and 75 non-active peptides (75p+75n) of E proteins were used as the training dataset in SVM learning; the remaining 26 active and 26 non-active peptides (26p+26n) were used as the test set.

**Table 1:** Experimentally validated peptide inhibitors of E proteins.

	Active peptides	Non-active peptides	Ref
**HIV**	**DCPNGPWVWVPAFCQAVGWG**	**SPLGFGSYTMTKIRDSLHLV**	[9]
**16p+31n**	**GPWVWVPAFCQAVGWGDPIT**	**ANGSRIPTGERVWDRGNVTL**	
	**LCDCPNGPWVWVPAFCQAVG**	**CGTCVRDCWPETGSVRFPFH**	
	**PNGPWVWVPAFCQAVGWGDP**	**CRANGSRIPTGERVWDRGNV**	
	**PTGERVWDRGNVTLLCDCPN**	**CSCRANGSRIPTGERVWDRG**	
	**RGNVTLLCDCPNGPWVWVPA**	**DLEAVPFVNRTTPFTIRGPL**	
	RIPTGERVWDRGNVTLLCDC	**ELSEWGVPCVTCILDRRPAS**	
	TLLCDCPNGPWVWVPAFCQA	**ETGSVRFPFHRCGTGPRLTK**	
	WDRGNVTLLCDCPNGPWVWV	**GAPASVLGSRPFDYGLKWQS**	
	WVWVPAFCQAVGWGDPITHW	**GLTGGFYEPLVRRCSELMGR**	
		**GNQGRGNPVRSPLGFGSYTM**	
		**GSRIPTGERVWDRGNVTLLC**	
		**HWSHGQNQWPLSCPQYVYGS**	
		**KCPTPAIEPPTGTFGFFPGV**	
		**LGSSDRDTVVELSEWGVPCV**	
		**LSCPQYVYGSVSVTCVWGSV**	
		**PFDYGLKWQSCSCRANGSRI**	
		**PPINNCMPLGTEVSEALGGA**	
		**QAVGWGDPITHWSHGQNQWP**	
		**RCGTGPRLTKDLEAVPFVNR**	
		**SKIDVWSLVPVGSASCTIAA**	
		**SPLGFGSYTMTKIRDSLHLV**	
		**SWFASTGGRDSKIDVWSLVP**	
		TCILDRRPASCGTCVRDCWP	
		TEVSEALGGAGLTGGFYEPL	
		TGTFGFFPGVPPINNCMPLG	
		TKIRDSLHLVKCPTPAIEPP	
		TTPFTIRGPLGNQGRGNPVR	
		VGSASCTIAALGSSDRDTVV	
		VRRCSELMGRRNPVCPGYAW	
		VSVTCVWGSVSWFASTGGRD	
	**LSGIVQQQNNLLRAIEAQQHLLQLTVWGIKQLQ**		[10]
	**SGIVQQQNNLLRAIEAQQHLLQLTVWGIKQLQARIL**		
	**NNLLRAIEAQQHLLQLTVWGIKQLQARILAVERYLKDQ**		
	YTSLIHSLIEESQNQQEKNEQELLELD		
	WMEWDREINNYTSLIHSLIEESQNQQEKNEQELL		
	**YTSLIHSLIEESQNQQEKNEQELLELDKWASLWNWF**		[8]
**DENV**	**RWMVWRHWFHRLRLPYNPGKNKQNQQWP**	**RWRHLKKMQRLQPRNPNWPGQFWVHYNW**	[17]
**5p+9n**	**FWFTLIKTQAKQPARYRRFC**	**MVIVQHQWMQIMRWPWQPE**	
	RQMRAWGQDYQHGGMGYSC	**QQCFRFPALRKKATYTRFWI**	
		**YPENLEYRVYITPHPGEEHH**	
		**EWSKHREGRWHTALTGATEI**	
		WHTVEPIVTEKDRPVNYEWE	
	**AWDFGSLGGVFTSIGKALHQVFGAIYGAA**		[19]
	**MAILGDTAWDFGSLGGVFTSIGKALHQVFGAIY**	**MVDRGWGNGCGLFGKGGIV**	[18]
		MVDRGWGNGCGLFGKGGIV	
		AWLVHTQWFLDLPLPWLPGADTQGSNWI	
DENV-DET	**PWLKPGDLDL**		[20]
	**AGVKDGKLDF**		
**2p+0n**			
**WNV**	**TFLVHREWFMDLNLPWSSAGSTVWR**	**VVDRGWGNGAGLFGKGSID**	[16]
**7p+13n**	TFLVHREWFMDLNLPWSSA		
	**DTRACDVIALLCHLNT**	**TGPEFPGRPTRP**	[18]
	**CDVIALLCHLNT**	**NTTHYRVIRLTIG**	
	**CDVIALLACHLNT**	**DTRACDVIALL**	
	**CDVIALLCHLNTPSFNTTHYRESWY**	**CDVIALLACHLNTPSFNTTHYRESWY**	
	CDVIALLCHLNTPSF	**TRACDVIALLECHLNT**	
		**DTRACDVIALLECHLNT**	
		**DTRACDVIPLL**	
		**CDVIALL**	
		**DTRAPLAI**	
		CDVIALLACHLNTPSF	
		CDVIALLECHLNT	
		DTRACDVIALLECHLNT	
**HCMV**	**WEIHHINKFAQAYSSYSRVIGGTVFVA**		[32]
**4p+0n**	**WHSRGSTWLYRETANLNAMLTITTARSKYPY**		
	**HFFATSTGDVVYISPFYNGTNRNASYFG**		
	FFIFPNYTIVSDFGRPNAA		
**HSV**	**KTTSSIEFARLQFTY**	**CPPPTGATVVQFEQP**	[31]
**4p+20n**	**GHRRYFTFGGGYVYF**	**CYSRPLVSFRYEDQG**	
	**HEVVPLEVYTRHEIK**	**DARDAMDRIFARRYN**	
	TTPKFTVAWDWVPKR	**DCIGKDARDAMDRIF**	
		**DDHETDMELKPANAA**	
		**DLKYNPSRVEAFHRY**	
		**DMELKPANAATRTSR**	
		**DNATVAAGHATLREH**	
		**DPKPKKNKKPKNPTP**	
		**EVIDKINAKGVCRST**	
		**EYPLSRVDLGDCIGK**	
		**FADIDTVIHADANAA**	
		**HVNDMLGRVAIAWCE**	
		**LEVYTRHEIKDSGLL**	
		**PVPFEEVIDKINAKG**	
		PYKFKATMYYKDVTV	
		TVSTFIDLNITMLED	
		APTSPGTPGVAAATQ	
		AYQPLLSNTLAELYV	
		CIVEEVDARSVYPYD	
**HSV-gH**	**AAHLIDALYAEFLGGRVLTTPVVHRALFYASAVLRQPFLAGVPSA**	**TWLATRGLLRSPGRYVYFSPSASTWPVGIWTTGELVLGCDAAL**	[26]
**3p+2n**	**GLASTLTRWAHYNALIRAF**	**RLTGLLATSGFAFVNAAHANGAVCLSDLLGFLAHSRALAG**	
	AAHLIDALYAEFLGGRVLTT		
**HSV-pTM**	**APSVFSSDVPSTALLLFPNGTVIHLLAFDTQPVAAIA**	**GPTEGAPSVFSSDVPSTALLLFPNG**	[27]
**6p+7n**	**TVIHLLAFDTQPVAAIAPGFLAA**	**APSVFSSDVPSTALLLFPNGTVIHL**	
	**SSDVPSTALLLFPNGTVIHLLAFDTKKKK**	**LFPNGTVIHLLAFDTQPVAAIAPGF**	
	**KKSSDVPSTALLLFPNGTVIHLLAFDTKK**	**GTVIHLLAFDTQPVAAIA**	
	STALLLFPNGTVIHLLAFDTQPVAAKKKK	**TVIHLLAFDTQPVAAIA**	
	KKSTALLLFPNGTVIHLLAFDTQPVAAKK	TVIHLLAFDTQPVAAIAPGFLAASA	
		SHVLTAPALTFNLTDFVPILALAGIQA	
**HSV-HB**	**VTVSQVWFGHRYSQFMGIF**	**FVLATGDFVYMSPFYGYRE**	[28]
**4p+4n**	**SVERIKTTSSIEFARLQFTYNHIQ**	**YGGSFRFSSDAISTTFTTN**	
	**PCTVGHRRYFTFGGGYVYF**	**YYLANGGFLIAYQPLLSNT**	
	YAYSHQLSRADITTVSTFI	FVRGHTGFVYCYGYTGFPR	
**HSV-HR**	**TARLQLEARLQHLVAEILEREQSLALHALGYQLAFV**	**LQLEARLQHLVAEILER**	[29]
**4p+9n**	**ALHALGYQLAFVLDSPSAY**	**YQFHLVLHEALRAQALSRQLILGRELAQELVAELAT**	
	**RARRSLLIASALCTSDVAAATNADLRTALARADHQKTLFWL**	**TSDVAAATNADLRTALARADHQKTLFWL**	
	AGDNATVAAGHATLREHLRDIKAENTDAN	**HATCSLAFALATSVALATRNDLLLRWAAARDAQTILSKRDRAGH**	
		**ATLREHLRDIKAENTDAN**	
		**TAAGDARANAVAKAGLHDLNIETDTERNH**	
		**VEGQLGENNELRLTRDAIE**	
		GENNELRLTRDAI	
		DVREEEQLGERATGLNLNI	
**HSV-gBh**	**SIEFARLQFTYNHIQRHVNDMLGRVAIAWCELQNHELTLWNEARK**		[30]
**11p+0n**	**SIEFARLQFTYNHIQRHVNDMLGR**		
	**VAIAWCELQNHELTLWNEARK**		
	**FARLQFTYNHIQRHVNDMLGR**		
	**FARLQFTYNHIQRHVRDMEGR**		
	**YNHIQRHVNDMLGR**		
	**YNHIQRHVNDMLGRVAIAWCE**		
	**YNHIQRHVNDMLGRVKKAWEE**		
	FARLQFTYNHIQRHVNDMLGRVAIAWCE		
	FARLQFTYNHIQRHVNDMLGRVKKAWEE		
	SIEFARLQFTYNHIQRHVNDMLGRVAIAWCELQNHE		
**JEV**	**ATSSANSKA**		[21]
**1p+0n**			
**RVFV**	**SGSWNFFDWFSGLMSWFGGPL**		[22]
**2p+0n**	**WNFFDWFSGLMSWFGGPLK**		
**HCV**	**MANAGLQLLGFILAFLGWIGAIVS**		[25]
**CLDN-1**	**MANAGLQLLGFILAFLGW**		
**11p+0n**	**LLGFILAFLGWIGAIVST**		
	**FILAFLGWIGAIVSTALP**		
	**AFLGWIGAIVSTALPQWR**		
	**GWIGAIVSTALPQWRIYS**		
	**GAIVSTALPQWRIYSYAG**		
	**MANAGLQLLGFILAFL**		
	MANAGLQLLGFILAFLGWIG		
	MANAGLQLLGFILAFLGWIGAI		
	MANAGLQLLGFILAFLGW		
**SARS**	**MWKTPTLKYFGGFNFSQIL**		[11]
**11p+6n**	**ATAGWTFGAGAALQIPFAMQMAY**		
	**GYHLMSFPQAAPHGVVFLHVTW**		
	**GVFVFNGTSWFITQRNFFS**		
	AACEVAKNLNESLIDLQELGKYEQYIKW		
	**PTTFMLKYDENGTITDAVDC**		[12]
	**YQDVNCTDVSTAIHADQLTP**		
	**QYGSFCTQLNRALSGIAAEQ**		
	IQKEIDRLNEVAKNLNESLI		
	**NGIGVTQNVLYENQKQIANQFNKAISQIQESLTTTSTA**	**FKLPLGINITNFRAILTAFS**	[13]
	**IQKEIDRLNEVAKNLNESLIDLQELGK**	**VLYNSTFFSTFKCYGVSATK**	
		**PALNCYWPLNDYGFYTTSGI**	
		**RDVSDFTDSVRDPKTSEILD**	
		SNNTIAIPTNFSISITTEVM	
		GIGVTQNVLYENQKQIANQF	
**FF-3**	**VEDTKIDLWSYNAELL**		[15]
**1p+0n**			
**PICV**	**GHTLKWLLELHFNVLHVTRHIGARCKT**		[14]
**5p+0n**	**HLIASLAQIIGDPKIAWVGK**		
	**HYNFLIIQNTTWENHCTYT**		
	**PGGYCLEQWAIIWAGIKCF**		
	LNLFKKTINGLISDSLVIR		
**HCV**	**VSGIYHVTNDCSNSSIVY**		[24]
**4p+0n**	**PSQKIQLVNTNGSWHINR**		
	**DYPYRLWHYPCTVNFTVF**		
	YLYGIGSAVVSFAIKWEY		

* The sequences in bold were used in the 75p+75n training set; the rest sequences were used in the 26p+26n test set.

#### SVM input features

Three SVM models were developed using different features as input descriptors, namely physicochemical properties (denoted as EAPphysico), amino acid composition (EAPcompo) and statistical scoring function amino acid composition (EAPscoring).

Knowledge-based statistical functions are rooted in the Bayesian (conditional) probability formalism and derived directly from properties observed in the known folded proteins [52–54]. In knowledge-based scoring function, it was presumed that averaging over different atom types in experimental conformations is an adequate representation of the random arrangements of these atom types in any compact conformation [55]. Because the three classes of E proteins have different structural folds, it is difficult to retrieve a structure-based feature that is relevant to their antiviral activities. Generally speaking, any property associated with folded proteins can be converted into an energy function [56]. Since amino acid sequence determines the structural folds and properties of proteins/peptides, we presumed that a sequence-based statistical scoring function averaging over different amino acid sequences exhibiting inhibitive activities is an adequate representation of the random combinations of all twenty amino acid exhibiting any activity. In this approach, a peptide sequence derived from E protein is represented by twenty features each corresponding to the propensity of observing each of the twenty natural amino acids to be either active or non-active. A vector space of twenty sequence-based statistical scores was used as the EAPscoring input entries in the SVM learning.

We also built a SVM model using physicochemical properties as input features. Because of the feature of membrane fusion process, it was suggested that functional regions in glycoproteins need to be solvent accessible, hydrophobic and flexible [57]. Actually the majority of known peptide entry inhibitors share a common physicochemical property of being hydrophobic and amphipathic with a propensity for binding to lipid membranes [58]. Therefore, here the properties of E peptide inhibitors were described by five physicochemical parameters: PI, MW, GRAVY index (positive and negative GRAVY values indicate hydrophobic and hydrophilic peptides, respectively), solvent accessibility (exposed or buried) and secondary structure features (propensity for adopting α-helix, β-sheet or turn structure). These physicochemical features were calculated for each of the peptides and used as the EAPphysico input entries in the SVM learning. A third SVM model EAPcompo was also built where the fractions of amino acids in a peptide were used as input features in the machine learning process.

#### SVM training

The SVM models were trained using the experimentally validated 75p+75n data sets. During 5-fold cross validation, the training set was randomly partitioned into four subsets with equal size of (15p+15n) and a remaining subset (15p+15n). Three SVM models were built using sequence-based statistical scores, physicochemical properties and amino acid composition, respectively. The performances of the three models are shown in Table 2. It can be seen that the EAPscoring model performed best among the three models during 5-fold cross validation. A "grid-search" combined with cross-validation was adopted to search for the optimal parameters -c and -g in SVM models [49]. The result of the grid search is shown in the support information (S1 File). It is shown that the performances of three EAP models during 5-fold cross validation have been improved significantly using the optimized parameters (Table 2).

**Table 2:** Performance of the AVPpred and EAPpred models training set V^75p+75n^.

Data set	Model	Sensitivity	Specificity	Accuracy	MCC
**EAP**	EAPphysico	79.37	71.26	74.67	0.5
**(default parameters)**					
	EAPcompo	66.99	87.23	73.33	0.5
	EAPscoring	100	92.59	96	0.92
**EAP**	EAPphysico	80	72.94	76	0.52
**(optimized parameters)**					
	EAPcompo	94.67	94.67	94.67	0.89
	EAPscoring	100	97.4	98.67	0.97

### Evaluation of the predictive efficiency of SVM models on independent test set

The performance of the SVM models was evaluated using an independent dataset of experimentally validated peptides that were not contained in the learning dataset (Table 1). In the EAPphysico model where physicochemical properties of peptides were used as input features, an accuracy of 65% with a MCC value of 0.31 was observed (Table 3). In the EAPcompo model where amino acid composition features were used, the predictive accuracy and the MCC value are slightly higher. When the sequence-based statistical function scores were used as input in the EAPscoring model, a remarkable accuracy of 92% was achieved with a MCC value of 0.84. Thus the sequence-based statistical scores developed in the present research are predominantly superior to the conventional physicochemical properties or amino acid decomposition features in identifying active peptides derived from enveloped proteins.

**Table 3:** Performance of AVPpred and EAPpred models on independent test set V^26p+26n^.

Model	Features	Sensitivity	Specificity	Accuracy	MCC
**AVPpred**	AVPmotif	100	50.98	51.92	0.14
	AVPphysico	72.22	61.76	65.38	0.32
	AVPcompo	63.16	57.58	59.62	0.20
	AVPalign	92.86	65.79	73.08	0.52
**EAPpred**	EAPphysico	68.18	63.33	65.38	0.31
	EAPcompo	72.41	78.26	75	0.5
	EAPscoring	92.3	92.3	92.3	0.84

### Comparison of the predictive efficiency of the AVP and EAP Models

AVPpred is a web server for prediction of the activities of general antiviral peptides (AVPs) based on a number of experimentally validated positive and negative data sets [43]. The peptide inhibitors employed in AVPpred target a variety of biological targets involved in virus infection. In contrast, the self-derived peptides of enveloped proteins being studied in the present research competitively bind to E proteins so as to mediate the virus fusion process. Because the self-derived peptides share similar mechanism of action, it is feasible to retrieve common features from them to build predictive SVM models. In order to evaluate the performance in predicting peptide inhibitors of the enveloped virus, we compared the AVPpred models with our EAPpred models using an independent 26p+26n dataset as test set. The results are shown in Table 3.

Four different features were employed in the AVPpred models, namely conserved motif search using MEME/MAST, amino acid composition, sequence alignment using BLAST and physicochemical parameters including secondary structure, charge, size, hydrophobicity and amphiphilic character [43]. When the AVPmotif model was used to predict the activities of the self-derived peptide inhibitors, it performed rather poorly with accuracy of 52% and MCC of 0.14. This is not surprising because AVPmotif was developed based on 20 general antiviral peptide motifs. However, the self-derived peptide inhibitors may not share a conserved motif with the general antiviral peptides since the latter interact with various biological targets with different mechanisms of action. In the AVPalign model, the peptide sequences were classified into active and non-active databases and the query peptide sequences were matched against the active and non-active databases using the BLAST program. Compared with AVPcompo and AVPphysico, AVPalign performed better with a predictive accuracy of 73% and MCC value of 0.52. Fusion mechanism is highly conserved among related viruses and entry of viruses into host cells has been inhibited by peptides derived from various regions of envelope glycoproteins [59]. Self-derived peptides would inhibit interactions of their original domain by mimicking its mode of binding to partner proteins [4]. Because similar sequences are often associated with similar structure and function, the sequence-based property AVPalign would account for the activities of the self-derived peptide inhibitors which regulate the virus fusion by mimicking the binding to E proteins.

In the AVPphysico model, 25 best performing physicochemical properties were selected out of the 544 properties to build the SVM model [43]. Antiviral peptide inhibitors are generally amphiphilic [60] and the activities of peptide entry inhibitors are dependent on their interfacial hydrophobicity [58]. Therefore we only employed five physicochemical properties reflecting hydrophobicity, solvent accessibility and secondary structure features as SVM input features. It was demonstrated that the accuracy and MCC of EAPphysico is comparable to that of AVPphysico model, indicating the five properties used in current modeling building are critical for their activities.

**Fig 1:**
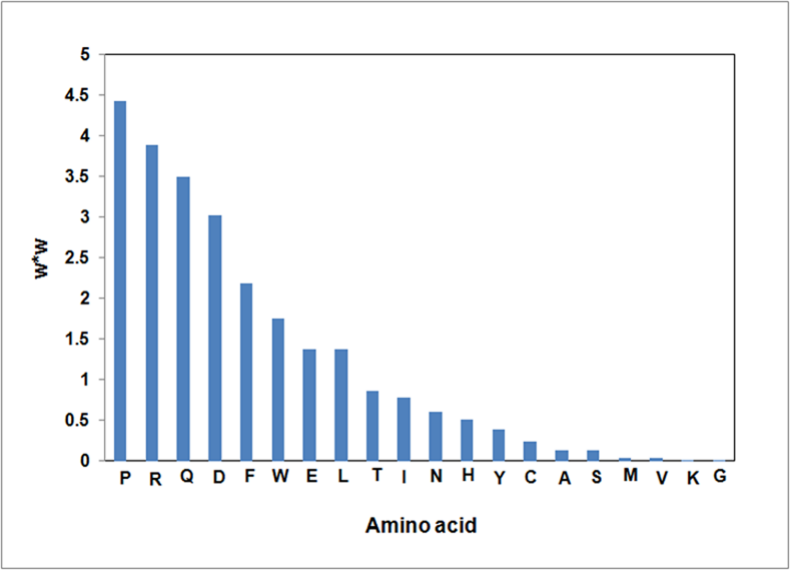
Feature ranking of the EAPcompo model. X-axis is the type of amino acid, Y-axis is *W* * *W*.

The MCC value of the AVPcompo models is 0.20, indicating that the antiviral activities of the peptides are related to amino acid composition. When the amino acid composition was used as input, the predictive accuracy of the EAPcompo model was higher than that of the AVPcompo model, indicating the peptide inhibitors of E proteins employed in the training set is sufficient to represent the contribution of amino acid composition to their inhibitive activities. In the EAPcompo model, the preference of the amino acid composition was ranked as: P, R, Q, D, F, W, E, L, T, I, N, H, Y, C, A, S, M, V, K, G (Fig 1). The role of arginine-arginine pairing and its contribution to protein-protein interactions has been investigated by computational approaches [61]. The higher abundance of R at protein-protein interfaces compared to K may be attributed to the formation of cation-π-interactions and the greater capacity of the guanidinium group in R to form hydrogen bonds (compared to K) [62–64]. Furthermore, it was suggested that the interface regions are enriched in aliphatic (L, V, I, M) and aromatic (H, F, Y, W) residues and depleted in charged residues (D, E, K) with the exception of arginine [62, 65–69]. This is in agreement with our amino acid composition analysis, where higher population of aliphatic Leu residue as well as aromatic residues Trp and Phe was observed, whereas positively charged Lys was hardly observed. The predominant occurrence of proline and glutamine residues is characteristic for the unique protein-protein interactions for E proteins. e.g. a conserved proline-rich motif was suggested to be engaged in monomer-monomer interactions in Dengue E proteins [70]. A conserved glutamine-rich layer is involved in the extensive H-bond network in HIV-1 gp41 E proteins [71]. Thus the preference of the amino acid composition identified from the EAPcompo model is generally in accordance with the predominant residues involved in protein-protein interactions, manifesting the amino acid composition of the self- derived peptide inhibitors are closely related to their potential activities in mediating the protein-protein interactions in the virus fusion process.

Because the antiviral activities of peptides are dependent on amino acid composition, we presume amino acid composition discriminated by the propensity of their activities would be an intrinsic feature in the self-derived peptide inhibitors which share a common mechanism of action. When statistical function scores were employed in the SVM model (EAPscoring), a remarkable predictive accuracy of 92% with an ideal MCC value of 0.84 was achieved, significantly better than any AVP models. The logarithm form of the discriminatory function (Eq 1) can be deemed as the pseudo energy of the system. In our previous study, we suggested that the stability of proteins is related to their *in situ* binding potential to the partner regions [72]. The prominent performance of EAPscoring model indicates the sequence-based stability feature of self-derived peptides may reflect their potential of binding to E proteins so as to regulate the virus entry process.

## Conclusions

We developed three SVM models using physicochemical properties, amino acid composition and statistical discriminative function as input features. The prediction accuracy and the MCC value of the EAPphysico model where five physicochemical properties were employed are comparable with the previous AVPphysico model where 25 physicochemical properties were used. The AVPcompo and EAPcompo models demonstrated that the activities of antiviral peptides are dependent on amino acid composition. A sequence-based scoring function was developed for the self-derived peptide inhibitors of E proteins. The outperformance of the EAPscoring models supports our hypothesis that an intrinsic feature, represented by the propensity of each amino acid for being active in self-derived peptides, is responsible for the activities of the peptides to regulate virus fusion by mimicking the binding to their accessory proteins. The sequence-based statistical scoring function would be useful in development of novel antiviral therapies to target the initial step of viral infection.

## Supporting Information

S1 File Parameters optimization by Grid-research combined with 5-fold cross validation. x-axis is log2^g^, y is log2^c^ and z-axis represents accuracy(%) (Figure A) Parameters Optimization for EAPphysico model. (Figure B) Parameters Optimization for EAPcompo model. (Figure C) Parameters Optimization for EAPscoring model.
(DOCX)
