# Supplementary material for: Identification of Peptide Inhibitors of Enveloped Viruses Using Support Vector Machine
Source: PLoS One. 2015 Dec 4;10(11):e0144171. doi: 10.1371/journal.pone.0144171 (PMC4670226; doi:10.1371/journal.pone.0144171)
Supplement: S1 File — x-axis is log2g, y is log2c and z-axis represents accuracy(%) (Figure A) Parameters Optimization for EAPphysico model. (Figure B) Parameters Optimization for EAPcompo model. (Figure C) Parameters Optimization for EAPscoring model. (DOCX) [file pone.0144171.s001.docx]

**Supporting information**

**S1 File.**

**Parameters optimization by Grid-research combined with 5-fold cross validation.** x-axis is log2^g^, y is log2^c^ and z-axis represents accuracy(%) **(Figure A)** Parameters Optimization for EAPphysico model. **(Figure B)** Parameters Optimization for EAPcompo model. **(Figure C)** Parameters Optimization for EAPscoring model.

**(Figure A)**

**(Figure B)**

**(Figure C)**
